# Wheat and chaff: the degree to which strategic management principles are integrated within corporate social responsibility reporting among large Canadian firms

**DOI:** 10.1186/s40991-022-00075-z

**Published:** 2022-11-03

**Authors:** Mark Fuller

**Affiliations:** grid.264060.6 0000 0004 1936 7363 St. Francis Xavier University, 3090 Martha Drive, B2G 2W5 Antigonish, NS Canada

**Keywords:** Corporate social responsibility reporting, Corporate disclosures, Strategic CSR, Strategic integration, Sustainability reports, Voluntary reporting, Canada

## Abstract

This empirical study examines the degree to which strategic principles are reflected in the corporate social responsibility (CSR) reporting practices among Canada’s largest corporations. In a two-phased approach, three time periods of corporate disclosure from 2016 to 2020 were studied. Using an organizational flowchart derived from the literature, CSR disclosures were organized according to six degrees of strategic integration. Analysis reveals a bimodal distribution of firms, with one mode representing firms with a fully integrated reporting framework, and another mode representing firms with the partial integration of strategic management principles. Inconsistent patterns of progress in reporting practices were discovered, with some firms demonstrating improved reporting practices while others stagnated or declined. Overall, a generalized improvement in strategic integration during our multi-year examination was observed. While this work has implications for corporate social responsibility theory, recommendations for CSR practitioners are also discussed.

## Introduction

Corporate social responsibility (CSR) reporting involves the collection, analysis, and dissemination of a firm’s social and environmental performance. Except where required by law or regulation, disclosure is customarily voluntary and involves both qualitative and quantitative managerial choices (Daub, 2007) that affect the quality and comparability of the reporting disclosures. Qualitative decisions include the determination of which information is materially relevant (Calabrese et al., 2016; Raith, 2022), the extent of management’s discussion and analysis of performance (Maroun, 2018; Neumann et al., 2011; Park et al., 2019), and the choices about which socio-environmental issues and stakeholder interests are prioritized (Kumar et al., 2021; Siltaoja, 2006; Thijssens et al., 2015). Quantitative decisions related to CSR reporting can disclose the firm’s current or ongoing socio-environmental performance (Dickson & Eckman, 2008; Saha, 2018; Veenstra and Ellemars, 2020), establish expectations or metrics for future performance (Al-Dah et al., 2018; Lin et al., 2017; Yu & Bondi, 2019), and identify variances between intended and realized performance (Mintzberg & Waters, 1985; Vitolla et al., 2017). Collectively, these decisions can provide strategic benefit to the firm, including reputation enhancement (Huang & Wang, 2022; Saeidi et al., 2015; Siltaoja, 2006), providing a source of environmental or social differentiation compared to rivals (Duanmu et al., 2018; Linder et al., 2014; Zhang et al., 2020), and supplementing the firm’s competitive advantages (Donnelly and Wickham, 2021; Porter & Kramer 2006; Saeidi et al., 2015). Yet not all CSR reporting activities in Canada are created equally, with firms engaged in voluntary disclosure free to determine the frequency of disclosure, the use of either idiosyncratic or externally developed reporting frameworks through which disclosure takes place, which if any metrics to use, and what information merits disclosure (Roca & Searcy, 2012; Coulmont et al., 2022). Research on the form and processes through which corporate social responsibility reporting takes place is therefore important if we are to critically assess a firm’s historical and comparative performance across different social and environmental indicators while deepening our understanding of the firm’s impact upon society.

The purpose of this empirical study is to examine the degree to which firms integrate strategic management principles within their CSR reporting, so that we can better understand the breadth of integration from highly integrated firms (“the wheat”) to those firms that have little or no integration (“the chaff”). We began our work with a preliminary qualitative examination of the sustainability reports from 47 large Canadian firms for the 2018 reporting period. Arising from this analysis, a classification flowchart was developed to assess sustainability disclosures according to six degrees of strategic integration. Applying this tool to the data set, a bimodal distribution was discovered in relation to the different levels of strategic integration, contrary to the expectation of a normal distribution throughout the various sustainability reports. Repetition analysis with the addition of two more reporting periods, 2016 as a preceding comparator and 2020 as a succeeding comparator, was utilized to examine longitudinal trends. Among the findings, we discovered that the frequency of non-reporting firms declined over time. It was further discerned that there was a general pattern of improvement in strategic integration coupled with less variability in reporting practices. That said, not all firms demonstrated continual improvement in their implementation of strategic CSR reporting practices: progression from lesser degrees of strategic integration to greater degrees was neither linear nor consistent from year-to-year. Additionally, it was found that identical bimodal distributions were present in all three time periods studied, the underlying cause of which merits further study.

This article contributes to the literature at the intersection of corporate social responsibility reporting and strategic management. The flowchart arising from this work facilitates the categorization of firms according to the degree to which strategic integration is incorporated within their sustainability reports. Our classification schema facilitates the longitudinal and cross-sectional comparison of firms’ CSR disclosures for researchers interested in comparative studies within the field. For CSR practitioners, we reflect on the need for firms to embrace greater consistency in the strategic management of their corporate social responsibility activities.

## Literature review

### The theoretical and philosophical contexts of CSR reporting

Much like the broader field of CSR in general, the topic of corporate social responsibility reporting has been closely associated with two theoretical traditions in the strategic management literature, stakeholder theory and the resource-based view (Köseoglu et al., 2021), of which the former has played a particularly influential role. Indeed, action by external stakeholders and managerial perceptions toward them have shaped and continue to influence the evolution of CSR disclosure (Diouf & Boiral, 2017; Hahn & Kühnen, 2013; Torelli et al., 2020). Irrespective of these differences in theoretical lenses, academics have embraced a shared epistemology, with researchers frequently drawing upon annual reports (Bansal, 2005; Donnelly & Wickham, 2020; Milne & Adler, 1999), a variety of standalone social responsibility and non-financial disclosures (Campopiano & Massis, 2015; Lock & Seele, 2016; Mazzotta et al., 2020), and environmental, social and governance (ESG) data (DesJardine et al., 2021; Roohani et al., 2009) as the foundations of knowledge for their studies. The use of sustainability reports to document CSR activities has increased in response to firm recognition of a plurality of stakeholder interests, and that these interests extend beyond those served by the regulatory reporting requirements of the firm (Neville & Menguc, 2006; Sen et al., 2006; Sweeney & Coughlan, 2008), producing an increasing variety of CSR disclosures including public accountability statements, codes of conduct, diversity and inclusion reports, emissions inventories, and environmental footprint reports for both stakeholders and researchers to assess and critique.

### Ontological differences and methodological approaches concerning CSR disclosure

Within the literature, there are ontologically disparate perspectives on the interpretation of firms’ CSR disclosures. Some academics doubt the extent to which CSR disclosures accurately reflect the intentions and the actions of the firm, expressing scepticism as to the credibility of the reporting (Dando & Swift, 2003; Mazzotta et al., 2020; McGuire et al., 1988). Factors contributing to this schism are the voluntary nature of reporting in most jurisdictions, firms’ self determination of materially relevant information, and the absence of mandatory disclosure standards. Accordingly, there are no guarantees of consistency regarding the frequency of reporting, the structure and format of the disclosure, and the consistency of the information shared. Methodologically, content analysis remains a frequent method of choice for qualitative studies in CSR (Fifka, 2013; Gupta & Das, 2022; Wolfe, 1991), though inconsistencies in disclosure at the firm level and differences in regulatory regimes at the industry, sub-state, and national levels make more difficult the longitudinal studies of individual firms, sectoral studies within industries, and cross-national comparisons. Although the rise in ESG data sets and “eXtensible Business Reporting Language” (XBRL) information has facilitated the use of statistical analysis (DesJardine et al., 2021; Roohani et al., 2009; Waddock & Graves, 1997), the aforementioned problems of voluntary reporting, structural reporting differences, and self disclosure variability have made quantitative approaches to the field more burdensome. Adding to the complexity of CSR reporting analysis, is that dissemination is commonly associated with large firms (Cormier & Magnan, 1999; Fifka, 2013; Patten, 1991) that possess the internal resources sufficient to collect, compile, and disclose information concerning their social and environmental activities, making problematic the consideration and analysis of small and medium-sized firms. The propensity to report has also been found to vary by industry (Fifka, 2013; Morhardt, 2010; Patten, 1991;) and has frequently been positively associated with firm performance (Cormier & Magnan, 1999; Okafor et al., 2021; Tagesson et al., 2009), among other variables, including corporate culture (Adams, 2002; McMurtrie, 2005), media coverage (Bansal, 2005; Bewley & Li, 2000; Brammer & Pavelin, 2004), political exposure (Bewley & Li, 2000; Steurer & Konrad, 2009), and as a counter to the liability of newness (Wang & Bansal, 2012), to name but a few. While many prospective benefits await firms that engage in CSR reporting, these benefits rest on the assumption that the ontological context of the reporting is objectively understood and widely shared among the organization’s stakeholders (Burrell & Morgan, 1979; Morgan, 1986).

### The use of reporting frameworks in CSR disclosure

Managerial decisions concerning the type and use of sustainability reporting frameworks within CSR disclosure play an important role in determining the nature and extent of the academic critique. Some research has examined the role of a particular reporting framework, such as the Global Reporting Initiative (GRI), the UN Global Compact, or the AA1000 AccountAbility Principles (Mio, 2010; Podrecca et al., 2021; Schadewitz & Niskala, 2010). Analysis of these types of frameworks provide value-added benefit to researchers analyzing CSR performance over time, and across industry and national boundaries because of the consistency of the reporting structures with which firms disclose their CSR activities. In contrast, other researchers have employed case studies to study individual firms where the reporting frameworks may be secondary in nature to the diversity and depth of particular disclosures (Ansu-Mensah et al., 2021; Hart, 2013; Hsu et al., 2013). Researchers have found that the use of reporting frameworks also evolves over time. As Coulmont et al., (2022, 11) noted, evidence suggests firms may produce idiosyncratic CSR reports in their initial years of reporting before eventually switching to more widely accepted reporting methodologies.

Adoption of externally developed reporting methodologies having assisted researchers in exploring many aspects of firm-level CSR activity, including the roles of various stakeholders. Among the diversity of stakeholder interests considered are those of board members (Fuente et al., 2017; García-Sánchez et al., 2019; Valls Martinez et al., 2019), investors (Verbeeten et al., 2016), and the LGBT community (Parizek & Evangelinos, 2021). Academic analysis and critique have also been applied to a variety of topical issues, including the cost of capital (Weber, 2018; Xu et al., 2021), femininity and gender diversity (Gallén & Peraita, 2017; Issa et al., 2022; Valls Martinez et al., 2019), information assurance (García-Sánchez et al., 2022; Hickman, 2020), and water use (Kleinman et al., 2017). Despite the benefits that externally defined, well adopted reporting frameworks have made for both comparative and critical analyses, their use by firms has not always been consistent. For example, previous research has found that performance metrics identified within the GRI are not reported uniformly, with CSR reporting frequently disclosing some indicators more than others (Roca & Searcy, 2012). Our own review of the GRI-based literature found it placed greater emphasis upon the disclosure of firm-determined materially relevant information over other factors, such as transparency and inter-firm comparability, as reflected in the debate over the materiality principle which some critics contend has reduced firm accountability to external stakeholders (Machado et al., 2021; Torelli et al., 2020; Zharfpeykan, 2021). Further examination of firm reporting on CSR strategic goals, targets, and performance could enhance our understanding of what stakeholders and socio-environmental issues matter, the degree to which the firm’s activities affect these stakeholders, and aid in furthering comparative studies of CSR reporting across firms.

From our review of the literature and CSR disclosures, we find that CSR reporting frameworks can be categorized along two dimensions. The first dimension involves the origin of reporting structures, and ranges from externally developed reporting structures developed by outside third parties (Mio, 2010; Podrecca et al., 2021; Schadewitz & Niskala, 2010) to internally developed reporting structures that are idiosyncratic to an individual firm, such as Coulmont et al., (2022) noted for firms in their early years of CSR disclosure. The second dimension is an indicator of the breadth of coverage in disclosing information that is materially relevant to a range of stakeholders, with holistic reporting frameworks being more inclusive of a range of issues and stakeholder interests than reporting methodologies which are more selective in nature. Externally developed, holistic frameworks are those that originate from a third party, are typically systematic in nature, and cover a broad array of reporting topics. These have been the focus of much research on CSR reporting (Machado et al., 2021; Milne & Gray, 2013; Roca & Searcy, 2012). Internally developed, selective CSR disclosures have been less well studied (Ansu-Mensah et al., 2021; Hart, 2013; Hsu et al., 2013), in part because evidence suggests firms with established CSR reporting practices adopt reporting structures that are the norm in their industry, but do not necessarily move beyond the minimum disclosure practices established by the reporting framework (Coulmont et al., 2022).

### Strategic integration within existing CSR reporting frameworks

Less well documented in the CSR reporting literature is the degree to which strategic management principles have been integrated within firms’ sustainability reporting, among both internally developed, selectively disclosed reporters as well as firms employing externally developed, holistically inclusive reporting frameworks. In particular, there are five strategic management principles, fundamental in nature to the field of strategic management, whose integration within CSR reporting we argue is especially important. We define these as follows:

- CSR strategy: the stated goal or goals that the firm is seeking to achieve in terms of its socio-environmental activities in order to obtain a competitive advantage. These may be multi-pronged or narrowly focused, inclusive of a number of stakeholders or a select few, addressing a range of stakeholder issues or limited in scope. For example, BCE Inc. (2019, 89) has a multi-pronged climate change strategy defined as follows:

Mitigating climate change is about reducing the release of GHG [greenhouse gas] emissions that are warming our planet. There are many mitigation strategies, including implementing energy savings initiatives, such as retrofitting buildings to make them more energy efficient; adopting renewable energy sources like solar and wind; and helping customers to reduce their own carbon footprint, for example through the use of technologies as a substitute for transportation.

- CSR objectives: one or more specific, measurable and relevant steps that advance progress toward achieving a particular CSR strategy (Doran, 1981). If the objectives are appropriate and timebound vis-à-vis the CSR strategies, then when all objectives are completed, the firm should achieve the intended CSR strategy. At BCE Inc. (2019, 93), one of their climate change objectives is as follows: “Our near-term objective is to reduce the ratio of our Scope 1 and 2 GHG emissions (tonnes of CO2 equivalent) to our network usage… by 75% of our 2014 level by the end of 2020.”
- Metrics and targets: Metrics are the quantifiable measures through which progress toward CSR objectives may be assessed, while targets are the a priori established, specific, anticipated results that the firm is striving to achieve using the associated metrics. Using our previous example, BCE Inc.’s (2019, 93) GHG emissions metric is Scope 1 and 2 emissions in tonnes divided by network usage in petabytes. The company has established annual targets for GHG emissions using this metric.
- Variance analysis: management statements that assess and disclose any factors which explain why the firm under or over-achieved a particular target. Understanding the rationale for divergent performance is necessary to undertake corrective action in order to improve the accuracy of future performance. For example, in 2018 BCE Inc. (2019, 22) had an emissions reduction of 73% from a base measure in 2014. GHG emissions were reduced 8% during the previous year and the company was 2% away from achieving its 2020 target, results that were verified by PricewaterhouseCoopers LLP.
- Future action: disclosures that detail forthcoming actions in pursuit of a CSR strategy, corrective action that the firm intends to pursue in order to address divergent performance, and forward-looking statements that signal impending activities. Returning to our BCE Inc. example (2019, 93), the company established future GHG targets for 2019 and 2020 and is working with the Carbon Disclosure Project and its partners to establish future emissions reductions objectives.

Some of these principles have been addressed more thoroughly than others; indeed, many articles have explored different aspects of CSR strategic formulation (Awaysheh et al., 2020; Donnelly & Wickham, 2020; Havlinova & Kukacka, 2021). Less attention has been given to the role of variance analysis on CSR-related managerial action (Dutta et al., 2013; Huang and Watson, 2015) and commitments to future action arising from past performance (Clarkson et al., 2013; Huang and Watson, 2015). While each of these principles is important in their own right, it is when we consider them in their totality as part of a feedback loop, embedded within the firm’s CSR disclosure practices, that we can separate the wheat from the chaff and identify those firms which have a more integrated strategic orientation within their socio-environmental reporting.

## Research methods and findings

This research project consisted of two phases. In Phase 1 of this research project, we collected CSR reports from 2018 for some of Canada’s largest firms in order to examine the degree of integration for the principles of strategic management discussed in our literature review: CSR strategies, objectives, metrics and targets, variation analysis, and future intended actions. We developed an organizational flowchart to categorize the CSR reporting activities of these firms in order to better understand the phenomena in question (Smith, 2002). The Phase 1 findings discussed below led us to wonder whether our results were anomalous or consistent with other reporting years. Accordingly, in Phase 2 of the study we examined the same firms’ reporting disclosures for 2016 and 2020 as a basis of longitudinal comparison, again using our classification flowchart. Our findings were consistent in both phases, across all three reporting periods, resulting in a number of implications for researchers and practitioners. Within this section, we describe our sample and our sampling rationale, research processes, and findings for both phases.

### Phase 1 sample and data collection

In exploring the degree of integration of strategic management principles within the sustainability reporting processes of firms, we chose to focus our research on the Canadian context because of its favourable regulatory regime that has positively affected CSR disclosure (Cormier & Magnan, 2007; Gallego-Álvarez & Pucheta-Martínez, 2021; Li & McConomy 1999), because Canada’s voluntary disclosure regime enables firms to chose the reporting frameworks best suited to their interests (Coulmont et al., 2022), and because Canadian-situated research specific to CSR reporting has been less addressed within the North American context (Fifka, 2013), albeit with greater interest in more recent years (Coulmont et al., 2022; Searcy et al., 2016; Thorne et al., 2017).

Previous studies have shown that firm size has been positively associated with CSR engagement (Alnajjar, 2000; Fifka, 2013; Patten, 1991), suggesting that larger firms are more likely to engage in CSR disclosure than small to medium firms because of the cost and resource requirements involved in developing and implementing a social responsibility strategy. In seeking a transparent source for our data that was publicly available without special subscription (Rahman and Post, 2010), and in keeping with previous Canadian research (Neu et al., 1998; May & Khare 2008; Zaichkowsky, 2014), we employed the Globe and Mail’s Report on Business database of the top 1,000 Canadian firms (Globe and Mail, 2019) to identify the largest companies from which to draw our sample for the 2018 reporting year. Using four metrics of firm size – market capitalization, total revenues, net income, and total assets – we compiled separate rankings of the 100 largest firms for each of these measures, resulting in 157 firms that possessed one or more of these measures. We then cross-referenced this list to identify which firms were present in all four metrics in order to establish a robust cohort of large firms, to enhance the comparability of firms as per May & Khare (2008), and to achieve a manageable dataset for qualitative analysis purposes. The result was an initial data sample that consisted of 47 firms drawn from the following ten industries (figures in parentheses are the number of firms in the sample): airlines (1), auto parts (2), basic materials (5), energy (10), entertainment (1), financial services (12), grocery (3), health care (1), industrials (3), packaging & containers (1), restaurants (1), retail (2), technology (1), telecommunications (3) and utilities (1). A complete list of firms studied is included in Appendix 1. We next collected the publicly available sustainability reports for the 2018 reporting period for these firms to capture the most recent period of pre-pandemic corporate disclosures; this was chosen to minimize the potential impact of Covid-19 effects on CSR reporting frequency and quality.

### Phase 1 data analysis

Our study examines the degree to which firms report to external stakeholders on their performance at various stages in the strategic design-implementation process. This can include whether the firm discloses their sustainability strategies and objectives; their performance metrics, targets and the associated results; managerial analysis of variances from expectations; and any intention to revise their plans and processes to remedy past performance. Given that our interest was in these specific phenomena rather than developing a grounded theory approach to CSR reporting, we utilized an a priori coding scheme (Neuendorf, 2002) to create a dictionary of strategic management terminology related to our strategic management principles: strategies; objectives; metrics and targets; variance analysis; and future performance. Employing Nvivo 12 qualitative analysis software, we organized each firm as a separate case. As we read the CSR reports for each firm, we expanded our coding key to identify language variations that related to our strategy principles. For example, many firms provide commentary on intended future actions using a variety of diction. These comments are issued within the context of relevant securities regulations, and as a consequence, many firms issue cautionary advice for stakeholders when considering a firm’s forward-looking statements. The Royal Bank of Canada (2019, 2), one of the firms examined in this study, identifies such statements with the following guidance:

Forward-looking statements are typically identified by words such as “believe”, “expect”, “foresee”, “forecast”, “anticipate”, “intend”, “estimate”, “goal”, “plan” and “project” and similar expressions of future or conditional verbs such as “will”, “may”, “should”, “could” or “would”.

Using cyclical coding, we employed our expanded coding key in iterative reviews of the CSR documents in our data set until no new clauses and phrases were encountered (Saldaña, 2016). We then returned to previously coded documents to apply newly discovered coding terms. The use of cyclical coding techniques featuring both multiple reads of each report allowed us to develop a classification flowchart for assessing firms’ CSR documentation based upon the strategic management principles found in the literature.

### CSR strategic integration flowchart

Based upon the strategic management principles found in the literature, and in consideration of the Phase 1 qualitative analysis, we developed a flowchart using the five strategic management principles to serve as distinct levels of classification, each level reflecting an increasing degree of CSR integration with their strategic planning processes. Beyond a basic typology (Smith, 2002), the flowchart aids us and future researchers to systematically classify CSR reports to comparatively assess social disclosures while laying the groundwork for future analysis. While most documents disclosed CSR activity with varying levels of strategic integration, we did identify reports that were primarily narrative in nature, discussing various aspects of their CSR performance in a non-strategic and loosely integrated manner. We classified these as “Level 1” reports. These firms appeared challenged to describe their corporate social performance in ways that incorporated a strategic management orientation. Reporting features generally included the use of discourse with internal CSR champions, employee profiles that highlight desirable social or environmental behaviors, and placing a spotlight upon external outreach activities including partnerships or external recognition. A lack of quantitative performance metrics was common; where quantitative data was provided, it failed to include specific goals, targets, or timeframes.

In contrast, firms that produced CSR reports that clearly articulated one or more CSR strategies were awarded a “Level 2” rating. Some firms defined strategies that were broad and encompassing; other firms defined multiple strategies that were narrower in scope. In either situation, it was insufficient merely to allude as to the existence of these strategies; these strategies had to be clearly defined and externally communicated. Firms in this category may have clearly stated their strategic goals, however the documentary evidence fell short of describing the means through which these goals were operationalized, lacking the provision of measurable targets, defined time periods, and the processes by which firms might achieve them.

“Level 3” reports went further. Not only were CSR-related strategies clearly articulated, but the firm also delineated a series of business objectives based upon these overarching goals. Some firms employed a systematic approach to defining their objectives, elaborating upon their strategies by delineating their intentions over the next fiscal year. Other firms employed objectives that were more loosely coupled to aspects of corporate social responsibility. Some firms alluded to the existence of measurable performance metrics; others may have claimed ambiguously defined goals were achieved. However, firms in this category stopped short of committing to externally published performance targets, reporting on quantifiable performance results, or defining time periods through which goals were intended to be achieved.

“Level 4” reports had well-defined strategies and clearly articulated the objectives though which these goals were to be operationalized and achieved. These objectives included metrics by which environmental and/or social performance was to be assessed, and externally communicated the targets for what the firm was striving to achieve. The firm’s performance in relation to these targets was self-disclosed and may have been verified through a third-party auditing process, though this latter characteristic was not used as a requirement. Firms in this category either failed to address the reasons behind the firm’s CSR performance shortcomings or made vague and ambiguous declarations about improving future performance.

“Level 5” reports possess all the characteristics of “Level 4” firms but go further in their self reflection. These firms acknowledged their shortcomings while analyzing and discussing these variances in past environmental and/or social performance. The depth and the quality of the analysis varied widely, with some firms concentrating on aspects of their under-performance, whereas other firms undertook a more systematic review. More retrospective than prospective, firms in this category fell short of discussing specific changes in work processes, policies, or strategies required to improve their future performance.

**Fig. 1:**
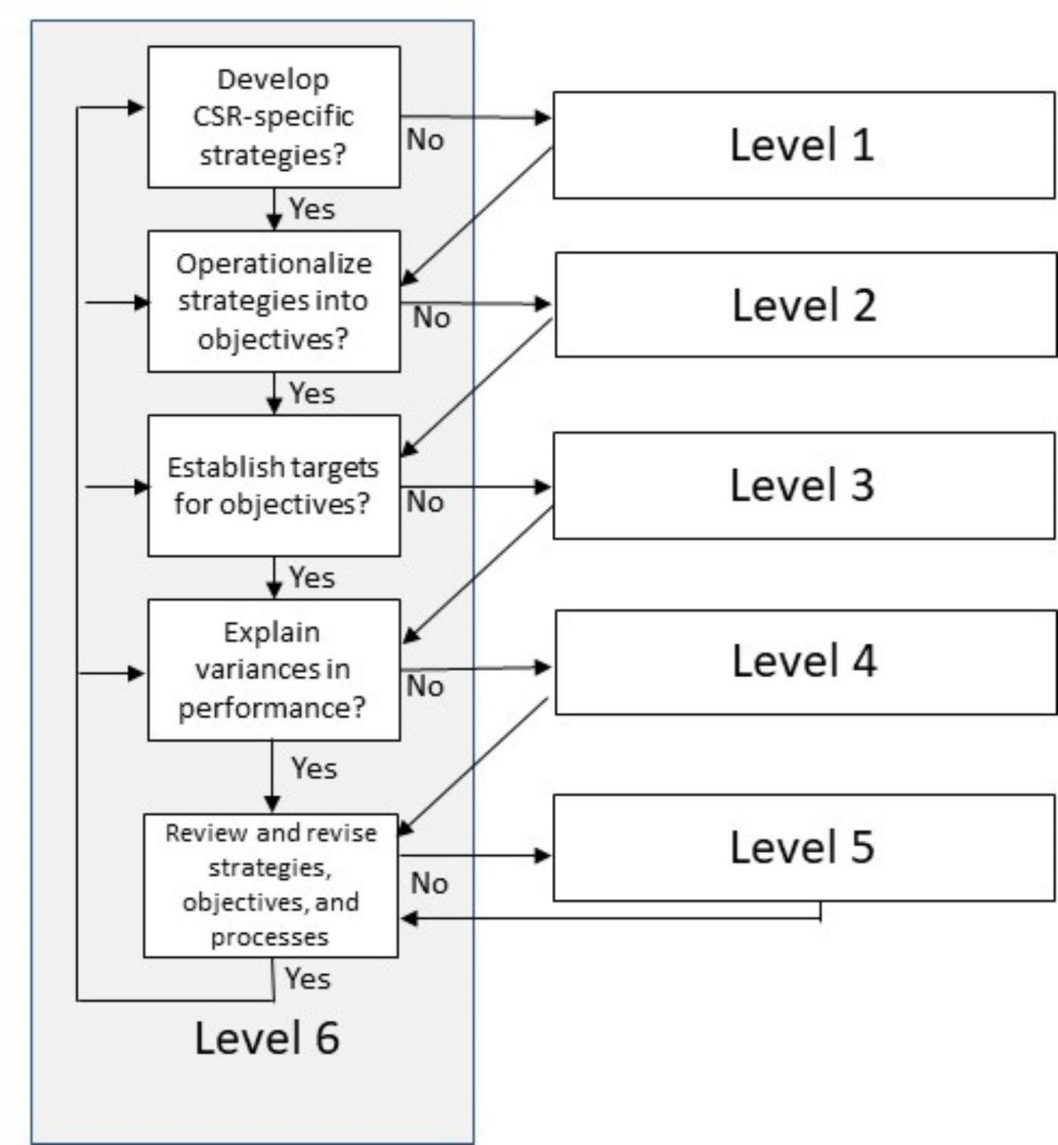
Strategic CSR Integration Flowchart

“Level 6” reports provided fully integrated strategic CSR plans. Management offered well-defined corporate social responsibility strategies that operationalized these into clearly articulated objectives. These objectives featured specific, measurable targets and deadlines for achieving each of these goals. When firm CSR performance underperformed expectations, the firm both explained the origins of the poor performance while also signalled the intention to revise the underlying processes and work activities, detailing how these shortfalls were to be remedied in the future. These different levels are shown diagrammatically in the Fig. 1 below:

### Phase 1 findings

Of the 47 identified focal firms, nine firms (19%) did not produce a publicly available 2018 CSR report. Firms were contacted by the authors about the missing reports. For firms that responded, the primary reasons given were that the firm had not yet produced its first CSR report in 2018 or that a subsequently published report retroactively covered multiple years, a phenomenon still seen occasionally in Canadian CSR disclosure practices. We found this level of non-reporting surprising, given that the literature suggests that large firms engage in CSR disclosure to a higher degree than small and medium-sized firms (Alnajjar, 2000; Fifka, 2013; Patten, 1991), and our data sample consisted solely of Canada’s largest, most profitable companies. This implies that future studies of CSR disclosure among Canadian small and medium-sized firms would encounter significant issues with data availability. We believe this level of non-disclosure reflects the still evolving and voluntary nature of corporate social responsibility reporting in Canada.

We then returned to our Phase 1 data and applied the cyclical coding process described previously for firms with publicly available CSR reports. As each report was read, we expanded our coding dictionary with new terminology as it was encountered (Saldaña, 2016). We applied our analytic framework, assigning an initial level to the company, which was reassessed following subsequent iterations of our coding process as our coding dictionary evolved, ensuring multiple reads and multiple assessments for each firm. To facilitate greater transparency (Rahman and Post, 2010), the final assessments for 2018 are provided in Appendix 1 and summarized in Table 1 below:

**Table 1:** Summary of Results (2018)

Level	No. of Firms	% Total	Cumulative %	Firms
0 (Non-Reporting)	9	19.15%	19.15%	Alimentation Couche-Tard, CCL Industries, CGI Group, George Weston, Linamar, Lions Gate Entertainment, Manulife Financial, Onex, Restaurant Brands International
1	3	6.38	25.53	Husky Energy, Keyera, Pembina Pipline
2	3	6.38	31.91	National Bank of Canada, Valeant Pharmaceuticals International, Waste Connections
3	4	8.51	40.42	Canadian Pacific Railway, Encana, Industrial Alliance, Magna International
4	11	23.40	63.82	Air Canada, Canadian Imperial Bank of Commerce, Canadian Tire, Canadian Utilities, Emera, Enbridge, Kinross Gold, Methanex, Metro, Power Corporation, Power Financial
5	2	4.26	68.08	Loblaw Companies, Sun Life Financial
6	15	31.91	100	Bank of Montreal, Bank of Nova Scotia, Barrick Gold, BCE Inc., CAE Inc., Canadian Natural Resources, Cenovus Energy, Hydro One, Lundin Mining, Rogers Communications, Royal Bank of Canada, Suncor Energy, Teck Resources, Telus, Toronto-Dominion Bank

**Fig. 2:**
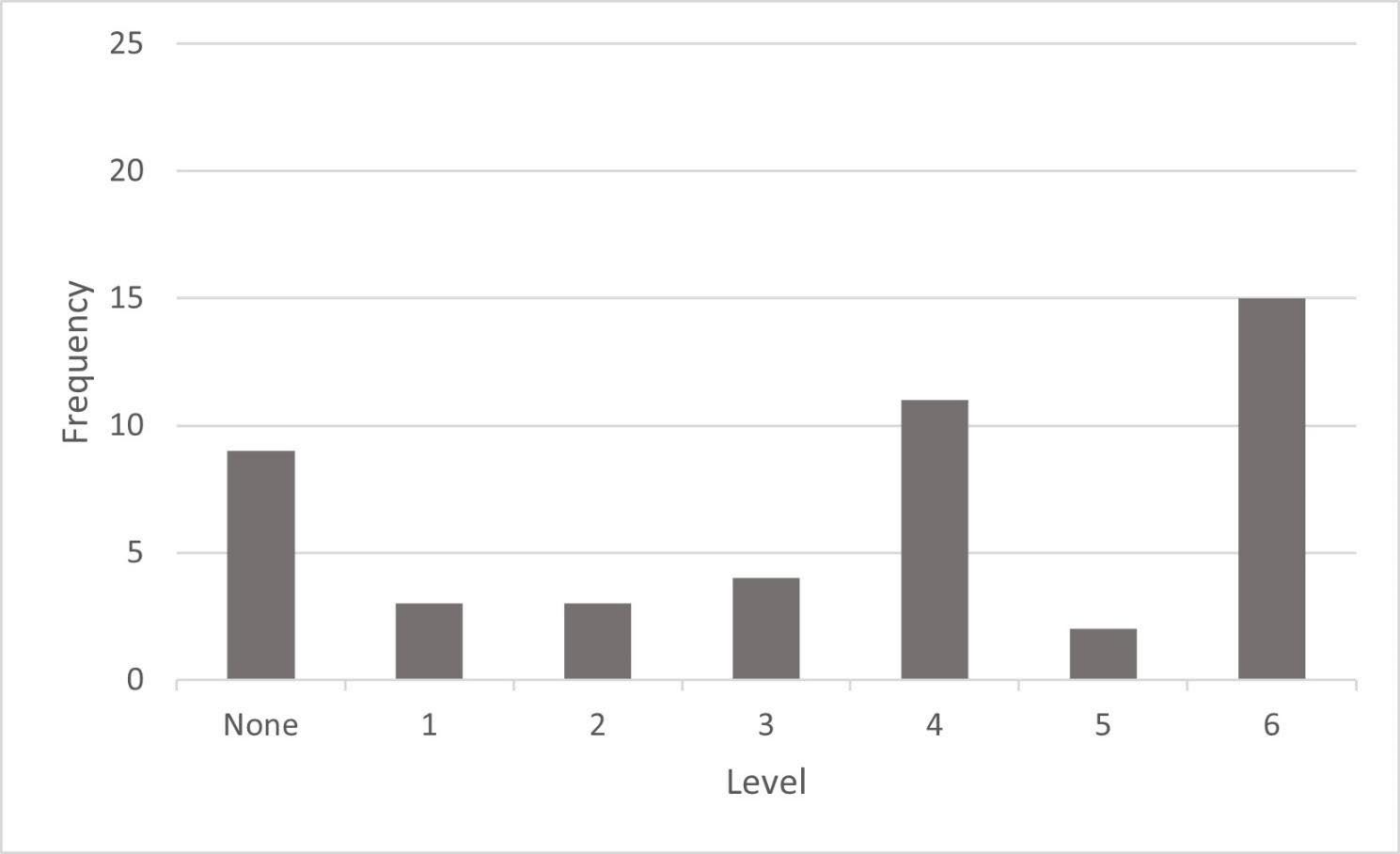
Frequency Distribution of Firms According to 2018 CSR Reporting

We categorized three firms as “Level 1” reporters based upon the available documentary evidence. These firms typically employed narrative discourse to describe their CSR performance, focusing on aspects of social, environmental, or governance activities without articulating an overall CSR strategy. Three additional firms were designated as “Level 2” reporters, providing one or more CSR-oriented strategies that emphasized social, environmental, or triple bottom line performance but did not delineate these into specific objectives. Four firms delineated their CSR strategies into clearly articulated objectives consistent with a “Level 3” designation; some may have alluded to the existence of performance targets but stopped short of externally documenting what these targets entailed. Meanwhile, 11 firms offered explicit performance metrics, provided specific targets for evaluating progress toward their CSR objectives, and documented their performance against these targets; these firms represented one of two peaks noted in the distribution of firms across the six degrees of strategic integration. However, these “Level 4” reporting firms failed to specifically analyze the reasons for performance variation, nor did they commit to specific actions for enhancing future performance. We identified two “Level 5” reporting firms that went further; they explored the factors that contributed to their underperformance but without explicitly stating what specific corrective action would be taken in the future. Fifteen firms achieved “Level 6” results – CSR strategies were articulated, objectives were defined, metrics and targets were provided, variation analysis was discussed, and specific future changes to work processes or business activities were described. Our Level 6 assessments represented the second of the two peaks noted in our distribution of firm reporting. We found this bimodal distribution interesting, as we had expected a frequency distribution similar to a normal distribution, with a central tendency (somewhere in the range of Level 3 or 4) and trailing off with few firms describing their CSR activities in an ambiguous manner (Levels 1 or 2), while others committed to variance analysis and specific business changes (Levels 5 or 6). The frequency distribution of our Phase 1 findings is illustrated in Fig. 2 below:

As we acknowledged earlier, we were surprised by the level of non-reporting firms in our study, in contrast to the literature which suggested an increase in the propensity of firms to disclose CSR-related information in response to stakeholder expectations (Neville & Menguc, 2006; Sen et al., 2006; Sweeney & Coughlan, 2008). With nine of the 47 firms not publishing a standalone sustainability report in 2018, this represented 19% of the firms that we identified. We did expect to find some “Level 1” firms to be slow to embrace sustainability, or at least to externally document their sustainability journey, as reflected in their use of environmental, social, or governance narratives. However, by 2018, we expected more firms to have established CSR strategies and operationalized these into business objectives, even if they did not develop performance targets and convey these to external audiences. Instead, we found a bimodal distribution, with 11 firms reporting at “Level 4” by including their CSR performance targets and results, and 15 firms landing in the “Level 6” category having analyzed their shortcomings and specified specific future actions to be undertaken. We draw from the Rogers (1962) diffusion of innovation curve to wonder whether early adopters to the value-added benefits of corporate social responsibility reporting might be reflected in the “Level 6” results while firms representing the early majority are presently situated further back at the “Level 4” designation. To explore these fluctuating findings, we decided to extend our study to include reporting periods before and after 2018 time period which comprised our Phase 2 data collection and analysis process.

### Phase 2 data collection

As a result of our unexpected findings in Phase 1, we extended our research endeavors to include a longitudinal analysis. We expanded our data collection for the 47 previously identified firms covering the reporting period of 2014 (the earliest period for which many CSR reports were still readily available) to 2020 (the most recent year for which data was available since there is a reporting delay between a firm’s CSR activities and the external reporting of said activities). Prior to 2016, we discovered a large degree of inconsistent reporting, with several firms not reporting at all, some firms reporting at irregular intervals, and the quality of reporting varying significantly among firms. The highly variable nature of the pre-2016 data undermined our confidence in drawing meaningful interpretations about the state of CSR reporting beyond that it was a nascent stage of development for many firms. Accordingly, we decided to compare our 2018 results to two data sets – sustainability reports from 2016 and from 2020 – to provide a two-year interval for comparative analysis purposes between our original data set, a predecessor data set, and the most recently available information in a successor data set. We attempted to collect publicly available CSR reports for all firms for the additional two reporting periods. Firms that did not publish a publicly available CSR report were contacted and asked if one existed and to supply it to the researchers where possible. We obtained CSR disclosures for 33 firms for 2016, 38 firms for 2018, and 43 firms for 2020, out of 47 firms for each sample period.

### Phase 2 data analysis

Our Phase 2 data analysis replicated the process from our previous research phase. We applied our classification flowchart to the additional two data sets, individually, beginning with the 2016 data and progressing to the 2020 data. We once again employed a cyclical coding process to assign initial assessments to CSR reports and then modified these assessments as our coding dictionary evolved within data sets. Firms for which CSR reports were unavailable received a zero score. Each firm for which a CSR report was obtainable was automatically awarded an initial “Level 1” score. Evidence of one or more CSR strategies increased that score to “Level 2”. Firms that demonstrated that they had operationalized their strategies into sustainability objectives had their score increased further to “Level 3”. The specification of performance metrics, targets and results were necessary to earn a “Level 4” standing. Evidence of variance analysis for firm under or over-performance elevated the score to a “Level 5” result. Firms that committed to specific future action – beyond ambiguous statements and legal definitions of forward-looking statements – were assigned a “Level 6” rating. We engaged in multiple reads and multiple assessments for each data set until no new additions to our coding dictionary were made, at which point moved on to the next data set.

### Phase 2 findings

We discovered some interesting trends in our longitudinal comparison of strategic integration within CSR reporting. First, we noted that there was an uptake in the number of firms publishing a dedicated sustainability report from the 2016 to the 2020 period, as summarized in Table 2 below (see Appendix 1 for firm-level data). Whereas 14 of 47 firms (29.79%) failed to produce a dedicated CSR report in 2016, that number was reduced to four firms (8.51%) by 2020. This is consistent with the extant literature that would suggest an increasing trend over time toward firm disclosure of CSR performance in response to stakeholder expectations (Neville & Menguc, 2006; Sen et al., 2006; Sweeney & Coughlan, 2008).

Our second observation suggests a general pattern of improvement over time. The number of firms producing sustainability reports without a strategically integrated framework (“Level 1”) was relatively consistent, at 2–3 firms per reporting period – but it is important to note that these were not necessarily the same firms in each period. For example, Pembina Pipeline did not publish a report in 2016, had published a narrative-style report (“Level 1”) in 2018, and a report that integrated the full-range of strategic principles in 2020 (“Level 6”), demonstrating remarkable improvement over a short period of time. In another case, Husky Energy achieved “Level 4” status in 2016, declined to “Level 1” in 2018, and rebounded to “Level 6” in 2020. In this study, we found 16 firms improved their assessments from 2016 to 2018, 24 remained stable, and six firms had a decline in their performance. From 2018 to 2020, 19 firms receiving higher ratings, 24 firms remaining stable, and just four firms receiving lower assessments. The sample means for the three reporting periods were 3.00, 4.00, and 5.00 and standard deviations of 2.51, 2.27, and 1.93 respectively. These results reflect a generalized shift toward improved strategic integration reporting with less variability in the assessments over time, as suggested in Table 2 below. However, as the firm level results in Appendix 1 suggest, CSR reporting quality from a strategic integration perspective is not assured. This suggests movement between the levels in neither uniform nor systematic in a stepwise function, as some firms moved upward or downward at differing and uneven rates of advancement or decline. Firms must work to both maintain and improve their performance from year to year, as the path forward is neither linear nor self evident for those firms who demonstrated reduced reporting quality.

**Table 2:** Summary of Results (2016 to 2020)

Level	2016	%	Cum. %	2018	%	Cum. %	2020	%	Cum. %
0	14	29.79%	29.79%	9	19.15%	19.15%	4	8.51%	8.51%
1	2	4.26%	34.04%	3	6.38%	25.53%	2	4.26%	12.77%
2	5	10.64%	44.68%	3	6.38%	31.91%	1	2.13%	14.89%
3	1	2.13%	46.81%	4	8.51%	40.43%	1	2.13%	17.02%
4	8	17.02%	63.83%	11	23.40%	63.83%	14	29.79%	46.81%
5	2	4.26%	68.09%	2	4.26%	68.09%	1	2.13%	48.94%
6	15	31.91%	100.00%	15	31.91%	100.00%	24	51.06%	100.00%
Mean	3.00			4.00			5.00		
Std Dev	2.51			2.27			1.93		

**Fig. 3:**
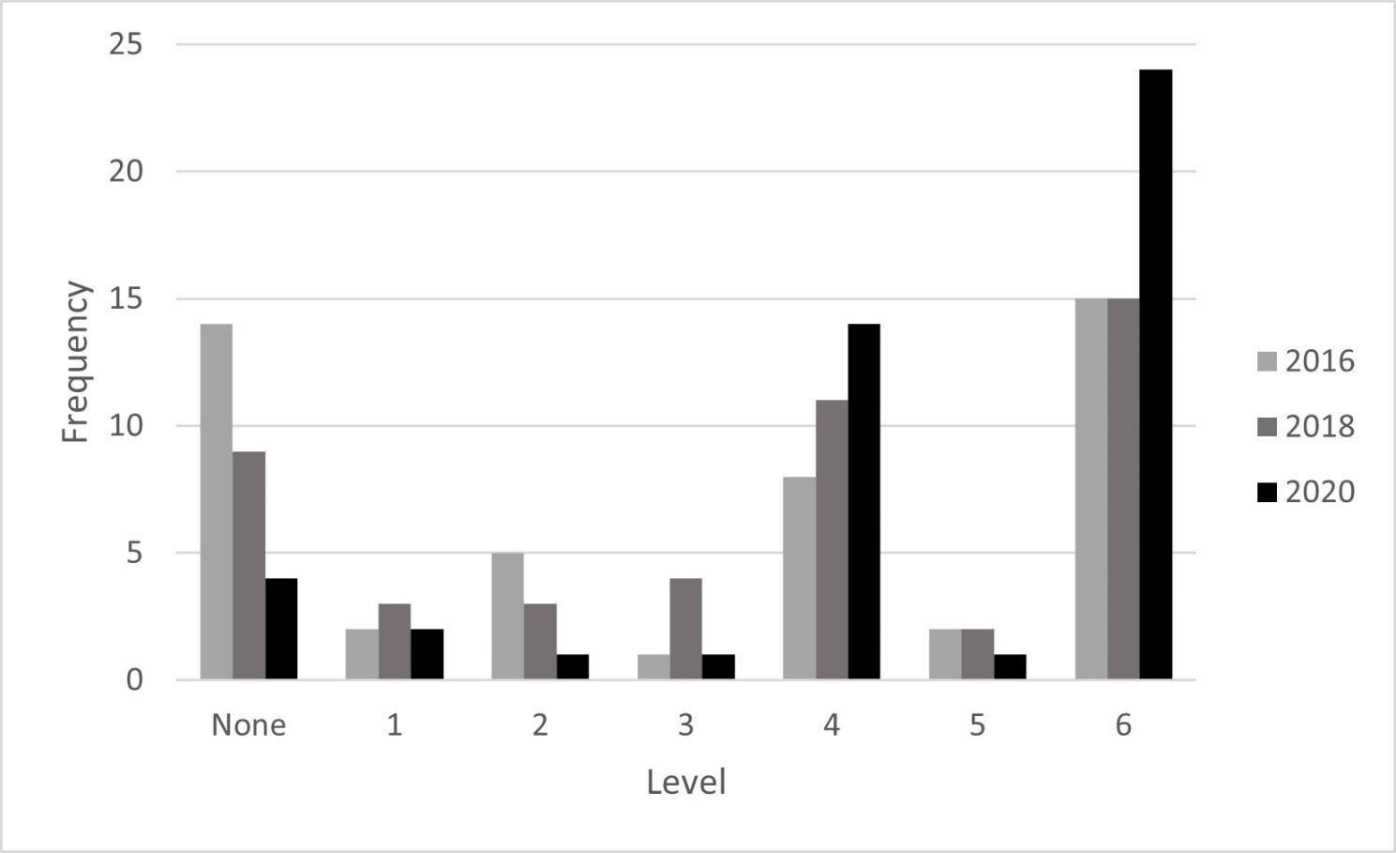
Multi-Year Frequency Distribution of Firms’ CSR Reporting

Our final observation relates to the bimodal distribution of CSR reporting illustrated below in Fig. 3. In our Phase 1 findings, we observed one mode at “Level 4” with firms reporting on performance metrics, targets, and results. The second mode occurred at “Level 6” that is associated with firms acknowledging underperformance and pledging specific actions in the future to improve the relevant processes or activities. Observations of these bimodal characteristics were consistently present in each of the additional reporting periods studied in Phase 2. Among the possible reasons why “Level 4” performance might be sticky for some firms while others skip “Level 5” and progress directly to “Level 6” are a lack of initial analysis for why CSR performance results fell short of expectations, a lack of internal leadership or external accountability motivating firms to improve their CSR disclosure (Diouf & Boiral, 2017; Hahn & Kühnen, 2013; Torelli et al., 2020), or a temporary period of commitment avoidance toward future organizational changes for which CSR performance may be uncertain (Coulmont et al., 2022). In fact, Coulmont et al., (2022) suggested firms switch from idiosyncratic reporting frameworks to externally developed frameworks in relatively short order but that their reporting disclosures can plateau within such frameworks.

## Discussion

This empirical study contributes to the CSR reporting body of knowledge by examining how firms integrate strategic management principles within their CSR reporting. We identified five principles from the literature – CSR strategies, objectives, metrics and targets, variance analysis, and future actions – and developed a flowchart for assessing the degree to which these principles were embedded within various corporate social responsibility reports. In a two-phase study, we studied CSR reporting by 47 large Canadian firms as the research suggests that larger firms have a greater propensity to engage in socio-environmental disclosure than small and medium-sized firms (Cormier & Magnan, 1999; Fifka, 2013; Patten, 1991). Our study of three reporting periods over five years produced three important findings. First, we observed an increase in Canadian firms’ propensity to report over the course of our study, consistent with expectations from the literature (Neville & Menguc, 2006; Sen et al., 2006; Sweeney & Coughlan, 2008). For the duration of our study, only one firm that previously released a sustainability report failed to produce one in a subsequent sample frame. Second, we observed an overall pattern of improvement across time periods. This was manifest by an increase in the overall mean scores for CSR reporting assessments and a decrease in the standard deviation across reporting periods. Our third observation was that the degree of strategic integration across time periods did not uniformly progress in a linear, consistent fashion. Rather, it produced repeating bimodal distributions peaking around “Level 4” and “Level 6” in each of the three reporting periods. This suggests that firms at a Level 4 designation have either some difficulty or some reluctance to enhance their performance further. These findings have important implications for the theory and practice of corporate social responsibility reporting.

### Implications for theory

In exploring the integration of strategic management principles within CSR reporting, our research explores important constructs related to sustainability disclosure. To preface this discussion, we note that our research methods involved cyclical coding (Saldaña, 2016) which required multiple reads and multiple assessments of various sustainability disclosures. During these evaluations, we observed differences in reporting quality (Daub, 2007) that were outside the scope of our research question. For example, firms using externally developed reporting structures, such as the GRI reporting standards, could differ significantly in the extent to which sustainability-related issues were discussed, analyzed, or became the focus of future action (Machado et al., 2021); similar differences were also noted by Roca & Searcy (2012) in the use of GRI metrics. As with Coulmont et al., (2022), we also noted sustainability disclosures that reported using idiosyncratic, internally defined reporting structures. Idiosyncratic methods are implicitly firm-specific, as each organization can self determine what information is materially relevant, what socio-environmental issues and stakeholder priorities to address, including what metrics if any to disclose. Our assessments of sustainability disclosures suggest firms which engage in idiosyncratic reporting are associated with a low level of strategic integration in their reporting; further, that the overall reporting quality was not particularly high. Thus, as we noted in our literature review, an important distinction should be made in terms of reporting inclusivity between holistically crafted and selectively drafted CSR disclosures (Di Vaio et al., 2022; Machado et al., 2021; Roca & Searcy, 2012). What this reinforced for us from a theoretical perspective is that reporting structure, reporting quality, and reporting inclusivity are different constructs from one another, with none serving as a dependent variable for the others in our view. Together, these three constructs may serve as antecedents of some other measure of reporting impact, a topic for future consideration.

A second implication for theory relates the use of idiosyncratic reporting structures to the bimodal frequency distribution we observed in each of our three data sets. Various authors have explored the adoption of external reporting frameworks (Coulmont et al., 2022; Fuente et al., 2017; Li & McConomy, 1999) but recent research suggested that their reporting disclosures can plateau within such frameworks (Coulmont et al., 2022). Likewise, we found evidence of firms switching from idiosyncratic to externally developed reporting structures, as well as evidence that some firms either struggle or choose to not move beyond the reporting of current performance against targets (Level 4) to variance analysis and commitment to specific future action (Level 6). Thus, it would seem there are two key decision points beyond whether or not to report: the first being whether to adopt an externally developed reporting structure (Fuente et al., 2017; Li & McConomy, 1999), and the second being a commitment to continual CSR improvement (Coulmont et al., 2022). If we term these decision points as a liability of adequacy, in which firms decide whether to report beyond the minimum norms of their industry, then overcoming this potential liability may require addressing one or more exogenous factors if the barriers to improved disclosure originate outside the firm or remedying issues of organizational inertia if the source is internal to the firm (Bettinazzi et al., 2020; Hannan & Freeman, 1989; Kelly & Amburgey, 1991). This latter possibility – that not every firm wants to be “the wheat” in terms of strategic integration and CSR disclosure and might rather be content as “the chaff” with little or no integration or continual improvement merits further research.

### Implications for practice

Regarding managerial practice, we would encourage a firm’s internal CSR champions to commit to the further integration of strategic management principles within their CSR disclosures. Whether this integration arises from the adoption of an externally developed reporting framework, such as the GRI, or idiosyncratic methods as described in this study, we believe reporting methods involving strategic integration assist both internal actors and external stakeholders to compare progress over time, assess the evolution of industry and national norms, and make informed managerial, investment, and relational decisions vis-à-vis the firm to support the continual improvement of CSR disclosures.

We would also advise that a wider application of strategic CSR throughout the firm’s social responsibility processes may supplement evidence that corporate social performance is positively associated with corporate financial performance (Flammer, 2015; Havlinova & Kukacka, 2021). This involves establishing CSR strategies, operationalizing these using well-defined objectives, choosing appropriate performance metrics, setting of targets, reporting on results according to these targets, explaining variation from these results, and describing future corrective actions to be implemented. Beyond reporting, not only is commitment to strategic integration within a broader approach to CSR important, but the application of these principles needs to be consistent. However, our research has indicated that not all firms progress in an ever-evolving fashion from less integrated to more integrated strategic principles in their reporting; some firms stagnate while other firms either advance or regress in the extent of their integration. This raises concerns about the extent to which various firms are committed to self improvement in their CSR disclosures. We would expect firms with a high degree of strategic integration would be naturally inclined toward continual improvement because of the feedback processes associated with the strategic management principles we investigated, such as the setting of targets, the measuring of performance against these targets, the analysis of variation, and an expressed commitment to change. Such firms are “the wheat” in our data set. However, the liability of adequacy defined earlier may help explain why some firms stagnate or even regress in the degree of strategic integration within their CSR disclosure processes. These firms, “the chaff” in our data set, would be well advised to institutionalize a strategic approach toward CSR disclosure if continual improvement is to be viewed as meaningful and valuable for the firm to maintain its socio-environmental performance in the wake of internal or external changes.

### Future research directions and limitations

We view the future of CSR disclosure research as moving beyond the establishment of norms toward the understanding of variances, but that one can only achieve progress on the latter by securing the former. If corporate social responsibility is viewed as either a managerial or as a societal innovation, then it is reasonable to assume that there will be a diffusion of adopters for the innovation (Rogers, 1962), with some firms being innovators, some laggards, and many firms in between. Much has and may still be written about the state of any given industry or national collectivity of firms on the CSR adoption curve. Yet the reason the curve exists at all is because management has the discretion to opt out of responsible management practices (Huang & Wang, 2022; Laasch & Conway, 2015; Zharfpeykan, 2021), despite a number of these practices becoming institutionalized norms through regulatory and legislative changes around the world. Regulation therefore becomes a necessary means to widely expand participation in responsible management practices. However, if such regulation enables a comply-or-explain approach, then the problems associated with some firms being reluctant to adhere to CSR disclosure requirements would continue to persist, as MacNeil & Xiao (2006) found in relation to accounting standards compliance. One of our near-term future research objectives should therefore explore what forms of principles-based regulation are optimal to balance a high degree of strategic integration within CSR reporting while ensuring sufficient economic freedom of our firms to be independently managed and operated. Experimental and conceptual studies may be beneficial in this regard, and insightful too, particularly in that they are among the least frequently employed methodological approaches to CSR research. Indeed, as Hahn & Kühnen (2013, 9) have reported, less than 1% of empirical CSR research articles employ experimental approaches. Following the near-term onboarding on firms becoming engaged in responsible management practices, researchers with a longer-term research orientation would then be better situated to explore variations in stakeholder responsiveness to a variety of CSR strategies by having a plethora of strategically integrated CSR-oriented firms from which to study.

As we conclude this study, we wish to contextualize some limitations within the work. Our first caveat is with respect to boundary conditions (Makadok et al., 2018). Geographically, our data collection was limited to the Canadian context where a CSR-favourable reporting regime is predominant (Cormier & Magnan, 2007; Gallego-Álvarez & Pucheta-Martínez, 2021; Li & McConomy 1999). Our results are premised upon the findings from this context. While we believe national analyses of jurisdictions where mandatory reporting regimes are in operation would benefit from the application of our classification flowchart for multi-sectoral analyses, we would anticipate different frequency distributions, particularly the longer a mandatory reporting regime has been in place.

A second boundary is a metaphysical one, where we harken back to our literature review and the differences between the epistemological legitimacy of our data sources and their ontologically subjective interpretation. As some authors have suggested, there may be discrepancies between the social and environmental activities and impacts of the firm, selective reporting, and corporate disclosure (Dando & Swift, 2003; Mazzotta et al., 2020; McGuire et al., 1988). Accordingly, our study does not capture undocumented CSR-related activities, and the CSR activities which are disclosed are acknowledged as social constructions of their respective firms subject to the interpretivism of both the corporate authors and external readers. Embracing qualitative research using methodologically different tools, such as participant observation studies (Kumar & Prakash, 2017; Hatipoglu et al., 2019; Lauring & Thomsen, 2009) and critical discourse analysis (Kumar & Prakash, 2017sänen & Vanharanta, 2016) could help inform our field as to the validity and the significance of this issue as the basis of future scholarship.

Finally, we wish to acknowledge issues of classification inherent in any classification scheme. As Smith (2002) and others have identified, classification errors from both the schema itself, and from the application by researchers, can produce issues with inter-rater reliability. While precautionary methods were taken with this study, including multiple reads and multiple assessments via the use of a cyclical coding process (Saldaña, 2016), we acknowledge that the classification of CSR reports is inherently subjective, and with that subjectivity comes the possibility of differences in interpretation.

## Data Availability

The CSR reports used in this study are publicly available from the individual firms.

## Appendix 1: List of Firms and Ratings (2016–2020). The following list identified the 47 firms that we used as part of this study, sorted by industry

**Table:**

No.	Company	Industry	2016 Level	2018 Level	2020 Level
1	Air Canada	Airlines	6	4	6
2	Linamar Corp.	Auto Parts	0	0	3
3	Magna International	Auto Parts	0	3	4
4	Barrick Gold	Basic Materials	6	6	4
5	Kinross Gold	Basic Materials	4	4	6
6	Lundin Mining	Basic Materials	5	6	6
7	Methanex Corp.	Basic Materials	4	4	4
8	Teck Resources	Basic Materials	6	6	6
9	Canadian Natural Resources	Energy	2	6	6
10	Cenovus Energy	Energy	6	6	6
11	Emera Inc.	Energy	4	4	6
12	Enbridge Inc.	Energy	6	4	6
13	Encana Corp.	Energy	0	3	6
14	Husky Energy	Energy	4	1	6
15	Hydro One Ltd.	Energy	6	6	6
16	Keyera Corp.	Energy	0	1	1
17	Pembina Pipeline Corp.	Energy	0	1	6
18	Suncor Energy	Energy	6	6	6
19	Lions Gate Entertainment	Entertainment	0	0	0
20	Bank of Montreal	Financial Services	2	6	6
21	Bank of Nova Scotia	Financial Services	4	6	5
22	Canadian Imperial Bank of Commerce	Financial Services	4	4	6
23	Industrial Alliance Insurance	Financial Services	2	3	6
24	Manulife Financial	Financial Services	0	0	0
25	National Bank of Canada	Financial Services	1	2	4
26	Onex Corp.	Financial Services	0	0	0
27	Power Corp. of Canada	Financial Services	6	4	4
28	Power Financial	Financial Services	6	4	4
29	Royal Bank of Canada	Financial Services	6	6	6
30	Sun Life Financial	Financial Services	4	5	6
31	Toronto-Dominion Bank	Financial Services	6	6	6
32	George Weston Ltd.	Grocery	0	0	0
33	Loblaw Companies Ltd.	Grocery	5	5	4
34	Metro Inc.	Grocery	4	4	4
35	Valeant Pharmaceuticals Int’l	Health care	0	2	2
36	CAE Inc.	Industrials	3	6	6
37	Canadian Pacific Railway Ltd.	Industrials	6	3	6
38	Waste Connections	Industrials	0	2	4
39	CCL Industries	Packaging & Containers	0	0	4
40	Restaurant Brands Int’l	Restaurants	2	0	1
41	Alimentation Couche-Tard	Retail	0	0	4
42	Canadian Tire Corp.	Retail	2	4	4
43	CGI Group	Technology	0	0	6
44	BCE Inc.	Telecom Services	6	6	6
45	Rogers Communications	Telecom Services	6	6	4
46	Telus Corp.	Telecom Services	6	6	6
47	Canadian Utilities	Utilities	1	4	4

## Abbreviations

CSR: Corporate Social Responsibility
ESG: Environmental, Social and Governance
GHG: Greenhouse Gas
GRI: Global Reporting Initiative
XBRL: Extensible Business Reporting Language
